# Case Report: A Rare Heterozygous *ATP8B1* Mutation in a BRIC1 Patient: Haploinsufficiency?

**DOI:** 10.3389/fmed.2022.897108

**Published:** 2022-06-16

**Authors:** Hao Bing, Yi-Ling Li, Dan Li, Chen Zhang, Bing Chang

**Affiliations:** ^1^Department of Gastroenterology, First Affiliated Hospital of China Medical University, Shenyang, China; ^2^Department of Gastroenterology, Shengjing Hospital Affiliated by China Medical University, Shenyang, China

**Keywords:** benign recurrent intrahepatic cholestasis, *ATP8B1*, haploinsufficiency, cholestasis, targeted therapy

## Abstract

Benign recurrent intrahepatic cholestasis (BRIC) is an autosomal recessive disorder characterized by recurrent cholestasis. ATPase class I, type 8B, member 1 (*ATP8B1*) encodes familial intrahepatic cholestasis 1 (FIC1), which acts as a phosphatidylserine reversing enzyme in the tubule membrane of hepatocytes to mediate the inward translocation of phosphatidylserine (PS). At present, dozens of *ATP8B1* pathogenic mutations have been identified that mainly cause BRIC1 and progressive familial intrahepatic cholestasis 1 (PFIC1). The diagnosis of BRIC1 is based on symptoms, laboratory tests, imaging, liver histology, and genetic testing. BRIC1 treatment seeks to prevent recurrence and reduce disease severity. At present, the main treatment methods include ursodeoxycholic acid (UDCA), rifampin, cholestyramine and haemofiltration, and endoscopic nasobiliary drainage (ENBD). Here, we report a 17-year-old patient with cholestasis who has a rare heterozygous *ATP8B1* gene mutation (p.T888K). The patient was treated with UDCA, glucocorticoids and haemofiltration, after which bilirubin levels gradually returned to normal. This case was thought to be caused by an *ATP8B1* heterozygous mutation, which may be related to haploinsufficiency (HI).

## Introduction

Familial intrahepatic cholestasis (FIC) is a group of autosomal recessive liver diseases characterized by intrahepatic cholestasis. FIC is a rare disease with an overall estimated incidence of 1 per 50,000 to 1 per 100,000 (1, 2). Benign recurrent intrahepatic cholestasis (BRIC) is characterized by recurrent jaundice, pruritus and malabsorption. The first appearance of jaundice in BRIC can occur at any age, usually < 20 years. BRIC-related symptoms generally last from weeks to months. Elevated serum bilirubin and BS levels were observed during cholestasis episodes, but gamma-glutamyltransferase (GGT) activity tended to be normal. BRIC episodes can be spontaneous or triggered by certain factors. Infection, pregnancy and medications are common triggers (3). BRIC does not progress to liver failure, and the associated symptoms resolve spontaneously. Symptoms may not occur in the intermittent period, and liver function and pathological findings are normal which increases the difficulty of diagnosis.

We report a case of jaundice. Laboratory tests showed significant increase in bilirubin. Biopsy and genetic testing were performed after common diseases were excluded. We found that the patient carried a rare *ATP8B1* gene mutation, which was consistent with the diagnosis of BRIC1.

## Case Report

**FIGURE 1:**
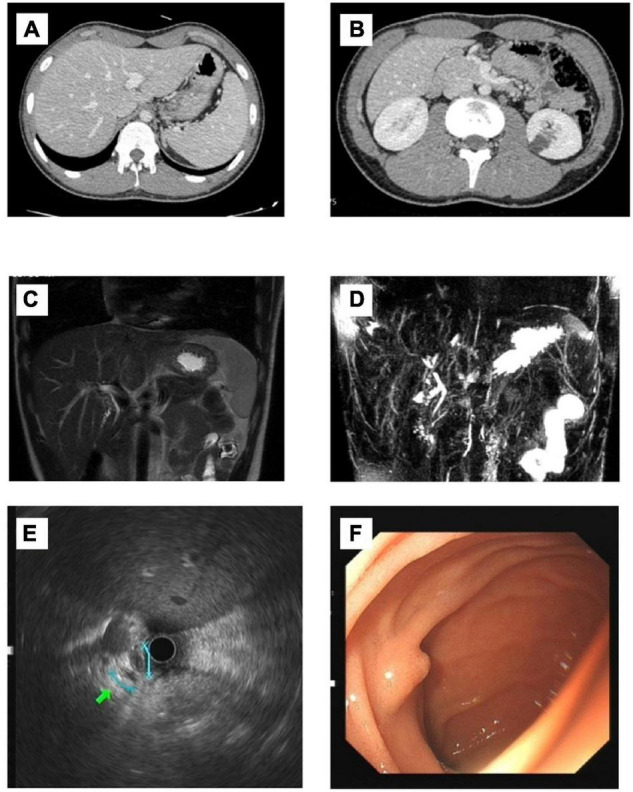
Imaging findings of the patient. **(A,B)** CT: No pathological findings were found. No dilatation of intrahepatic and external bile ducts was noted. There was no thickening or enhancement of extrahepatic bile ducts. The gallbladder is collapsed without bile filling; **(C,D)** MRCP: Possible stenosis at the beginning of the common hepatic duct. **(E)** Endoscopic ultrasonography: The extrahepatic bile duct was fine without dilation, and no definite obstruction was observed. **(F)** Gastroscopy: The size and morphology of the duodenal papilla were normal.

A 17-year-old boy presented with jaundice, white clay stool, nausea, and loss of appetite for 1 month. There was no history of rash 3 months before admission. Chinese medicine was externally applied, and the patient took anti-allergy drugs orally. Later, the rash improved, and fever occurred intermittently. The patient was a student with no history of other drug use, blood transfusions, allergies, smoking, or alcohol. His parents are in good health. Physical examination found that his skin and sclera were yellow. No other abnormalities were noted upon physical examination. The liver-related laboratory examination revealed cholestasis, and GGT levels were normal. Aspartate aminotransferase (AST), 36 U/L; alanine aminotransferase (ALT), 35 U/L; alkaline phosphatase (ALP), 157 U/L; Total bile acid (TBA), 150 μmol/L; total bilirubin (TBIL), 298.4 μmol/L; and direct bilirubin (DBIL), 227.4 μmol/L. Serological and laboratory results excluded autoimmune hepatitis, primary biliary cholangitis (PBC), viral hepatitis, Wilson disease and a1-antitrypsin deficiency (Table 1). Ultrasound examination showed no cholelithiasis or bile duct dilatation. The liver stiffness measurement was 11.4 kPa. Computed tomography (CT) shows no abnormality in the liver, and the gallbladder is collapsed without bile filling. Magnetic resonance cholangiopancreatography (MRCP) shows suspected stenosis at the beginning of the common hepatic duct. Endoscopic ultrasonography showed that the extrahepatic bile duct was normal without dilation, and no definite obstruction was observed. Gastroscopy showed the size and morphology of the duodenal papilla were normal (Figure 1).

**TABLE 1:** Laboratory data on admission at previous hospital.

Biochemistry		Reference range	Peripheral blood		Reference range
TP (g/L)	58.7	65.0–85.0	WBC (×10^9^/L)	5.92	4.0–10.0
Albumin (g/L)	32.7	40.0–55.0	Neutrophil (%)	47.9	40.0–75.0
TBIL (μmol/L)	294.8	3.4–20.5	Lymphocyte (%)	38.9	20.0–50.0
DBIL (μmol/L)	227.4	0.0–6.8	Monocyte (%)	10	3.0–10.0
AST (U/L)	36	15–40	Eosinophil (%)	2.2	0.4–8.0
ALT (U/L)	35	9–50	Basophile (%)	1.0	0.0–1.0
ALP (U/L)	157	45–125	Lymphocyte (%)	38.9	20.0–50.0
GGT (U/L)	19	10–60	RBC (×10^12^/L)	4.51	4.0–4.5
Urea (mmol/L)	3.68	2.85–7.14	Hb (g/L)	137	120–140
Cr (μmol/L)	56	29–104	Hct (L/L)	0.396	0.4–0.5
Cys-C (mg/L)	1.04	0.53–0.95	PLT (×10^9^/L)	310	100–300
	
Na (mmol/L)	136.5	137.0–147.0	**Coagulation**		
	
Cl (mmol/L)	103.7	99.0–110.0	PT (s)	13.5	11–13.7
K (mmol/L)	4.13	3.50–5.30	INR	1.02	0.9–1.1
CRP (mg/L)	< 1.00	0.00–5.00	PTA (%)	97.0	80.0–120.0
	
TBA (μmol/L)	150	0–10	**Serology**		
	
TSH (mIU/L)	1.137	0.35–4.94	ANA	(–)	(–)
FT3 (pmol/L)	2.48	2.63–5.7	AMA/AMA-M2	(–)	(–)
FT4 (pmol/L)	10.77	9.01–19.05	α1 globulin (%)	3.9	2.68–5.03
IgG4 (g/L)	0.301	0.049–1.985	γ globulin (%)	14.5	9.1–19.81
	
IgG (g/L)	10.18	7.00–17.00	**Viral markers**		
	
IgA (g/L)	2.01	0.70–3.80	Anti-HAV (S/CO)	0.11	0.00–0.80
IgM (g/L)	0.73	0.60–2.50	HBsAg (IU/mL)	0	< 0.05
Ferritin (μg/L)	722	30.00–400.00	HCVAb (S/CO)	0.28	< 1.00
Fe (μmol/L)	24.7	8.3–28.3	HEV-IgM (S/CO)	0.02	0.00–1.00
AFP (ng/mL)	< 0.91	0.00–7.00	Anti-CMV	(–)	(–)
CA19-9 (U/mL)	28.50	0.00–27.00	EBV DNA (copies/mL)	< 5.00E3	< 5.00E3
Ceruloplasmin (mg/L)	347.00	200.00–600.00	Anti-Sarkozy virus	(–)	(–)

*TP, total protein; TBIL, total bilirubin; DBIL, direct bilirubin; AST, aspartate aminotransferase; ALT, alanine transaminase; ALP, alkaline phosphatase; GGT, gamma-glutamyl transferase; Cr, creatinine; Cys-c, cystatin C; CRP, C-reactive protein; TBA, total bile acid; TSH, thyroid stimulating hormone; FT3, free T3; FT4, free T4; Fe, ferrum; AFP, alpha fetoprotein; CA 19-9, carbohydrate antigen 19-9; WBC, white blood cell; RBC, red blood cell; Hb, hemoglobin; Hct, hematocrit; PLT, platelet; PT, prothrombin time; INR, international normalized ratio; PTA. prothrombin time activity; ANA, antinuclear antibodies; AMA, anti-mitochondrial antibody; AMA-M2, anti-mitochondrial M2 antibody; HAV, hepatitis A virus; HBsAg, hepatitis B surface antigen; HCVAb, hepatitis C virus antibody; HEV, hepatitis E antibody; CMV, cytomegalovirus; EBV, Epstein-Barr virus; DNA, deoxyribonucleic acid.*

**FIGURE 2:**
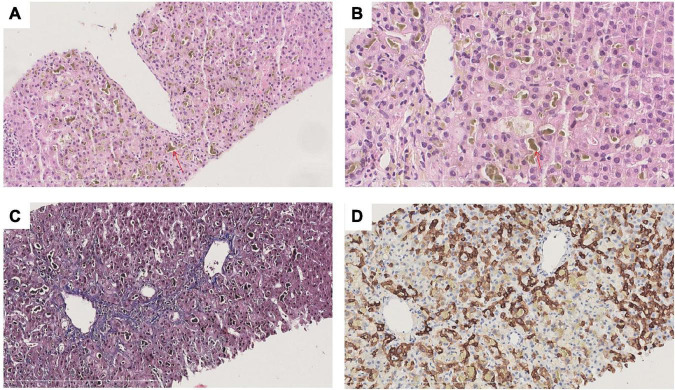
Histological findings of liver biopsy in the patient. **(A)** HE staining at 200× magnification; **(B)** HE staining at 400× magnification; **(C)** CK7 staining at 200× magnification; **(D)** Masson staining at 200× magnification. The lobule structure is clear. The main lesions were cholestasis with hepatocytes in central lobules II and III (red arrow), bile embolism with capillary bile ducts and cholestasis with Kupffer cells. No obvious inflammatory necrosis was observed in the lobules. No enlargement in the sink area or obvious inflammatory cell infiltration was noted. No interfacial inflammation was observed. A small bile duct can be identified. The epithelium of the bile duct is arranged in an orderly manner, and there is no bile duct reaction around the sink area. There was no fibrous tissue proliferation in the interstitium of the portal area, and the portal veins were discernible.

To clarify the cause of cholestasis, liver biopsy was performed and sections stained with hematoxylin-eosin (HE), Masson, mesh, PAS, DPAS, iron, CK7, CK19, CD10 and bile salt export pump (BSEP). Liver biopsy revealed a clear lobular structure. The main lesions were cholestasis with hepatocytes in central lobules II and III, bile embolism of capillary bile ducts and cholestasis with Kupffer cells, and positive staining for CK7 in some hepatocytes in the lobule (Figure 2). Pathological-clinical diagnosis is simple cholestasis. Genetic testing was performed on the patient and his parents. The patient’s genetic test revealed a rare missense mutation *ATP8B1* rs540027832 (chr18-55328450 c.2663C > A p. T888K NM_00560 3.4) (Supplementary Table 1), and genetic tests on his parents showed that the mutation was inherited from his father (Supplementary Figure 1).

The patient’s clinical manifestations and related examination results conformed to BRIC1. Drug-induced liver injury cannot be excluded. The patient was treated with glycyrrhizin, ursodeoxycholic acid (UDCA), glucocorticoids and haemofiltration. After 5 rounds of haemofiltration, the patient’s symptoms gradually improved, and the bilirubin index gradually decreased. After follow-up, bilirubin gradually decreased to normal within 3 months.

## Discussion

We report a patient carrying a rare heterozygous mutation of *APT8B1*, whose symptoms improved after drug therapy and blood purification. We searched some databases including Clin var, Leiden open variation database, NCBI Gene and gnomAD database. We found only one African male with the same mutation was included in gnomAD database. Besides, there has another variant p.T888M in the same position described, indicates that the mutation has more than one allele. *ATP8B1* rs540027832 p.T888M is also a rare mutation, with one European and one East Asian present in gnomAD database. *ATP8B1* rs540027832 p.T888K and p.T888M were present at frequencies of 0.000398% (1/251360 alleles) and 0.0007074% (2/282738 alleles), respectively, and never appeared in homozygous status in the gnomAD frequency database. Based on the clinical characteristics, pathological and genetic mutation changes, this patient was ultimately highly suspected of being BRIC1.

*ATP8B1* mutations can manifest as a range of diseases with BRIC1 and progressive familial cholestasis (PFIC)1 representing the two extremes of the phenotype. The development of PFIC1 in BRIC1 patients has been reported clinically (4). Intrahepatic cholestasis of pregnancy (ICP) is also associated with *ATP8B1* mutations (5). This patient may have an intermediate stages from BRIC1 to PFIC1. Due to its late onset and good results after treatment, we believe that the diagnosis of BRIC is the preferred choice, and the specific diagnosis still needs further follow-up and observation.

Clinical cases of BRIC1 caused by missense mutation of *ATP8B1* have been reported. Cases (6, 7) from Korea and Japan with novel heterozygous mutations leading to BRIC1 were reported, showing that BRIC1, as an autosomal dominant disease, may also caused by *ATP8B1* heterozygous mutations. Another report has summarized the genetic mutations in the 180 families of BRIC1 or PFIC1 cases (8). The results showed that missense mutations were more common in BRIC, while nonsense, frameshift, and large deletion mutations were more common in PFIC. We have not found any cases of disease caused by this mutation in the past. Our genetic test results can cover 99.9% of the target area. Undetected variation outside the detection range cannot be excluded. We focuses on summarizing the pathophysiology and clinical manifestations of BRIC1 and perspectives for the future development direction.

**FIGURE 3:**
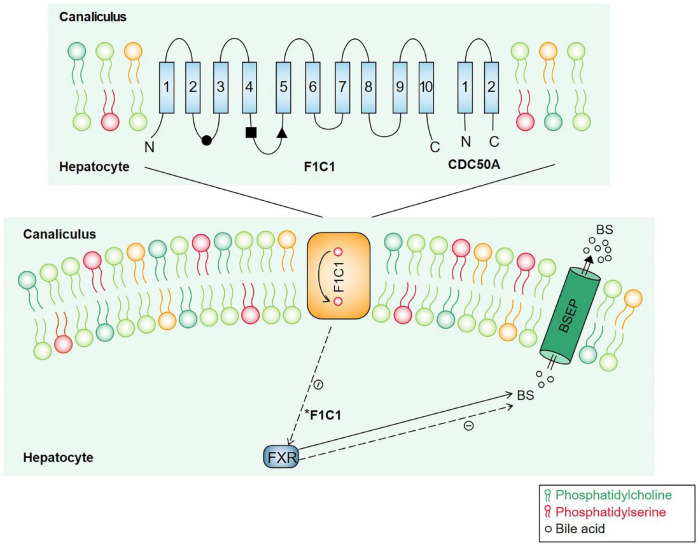
Molecular mechanisms underlying cholestasis associated with *ATP8B1* deficiency. ATP8B1 consists of 10 transmembrane segments, and ATP8B1 and CDC50A assemble to form a heterodimer complex that participates in PS flipping. BESP is a canalicular bile salt transporter. FXR is a nuclear receptor involved in regulating bile acid metabolism. When ATP8B1 is defective, the nuclear translocation of FXR is disrupted, and BSEP expression on the hepatic duct membrane is reduced due to its transcriptional inhibition. *Dotted line indicated a negative effect.

*ATP8B1* is located in the tubular membrane of hepatocytes, and the encoded FIC1 protein has 10 transmembrane domains that participate in the transport of phospholipids in the membrane and turn phosphatidylserine (PS) from the outer lipid leaflets back to the inner lipid leaflets of the tubular membrane to maintain the asymmetry and fluidity of the tubular membrane (9). *ATP8B1* and the transmembrane protein CDC50A form a heterodimer complex that promotes its correct transport to the plasma membrane (10). *ATP8B1* mutations can affect its stability and its interaction with CDC50A (11, 12). *ATP8B1* mutations reduced BSEP activity and impaired bile excretion by affecting PS turnover (13). In *ATP8B1* deficient patients, the nuclear translocation of the Farnesoid X receptor (FXR), a transcription factor that controls bile acid homeostasis, is disrupted, and BSEP expression on the hepatic duct membrane is reduced due to its transcriptional inhibition (14, 15) (Figure 3).

The pathological findings of BRIC1 are non-specific. At the onset, BRIC1 presents as centrilobular cholestasis, in which bile deposits are noted in tubules, hepatocytes and Kupffer cells, and bile embolism may occur in the tubules (16). *ATP8B1* detection is particularly important for the diagnosis of BRIC1. In response, resequencing chips have been developed specifically to look for genetic syndromes of cholestasis, which can aid in diagnosis (17). Luketic and Shiffman (16) proposed BRIC diagnostic criteria. For patients with intrahepatic cholestasis of unknown cause, when the known common causes cannot explain the patient’s condition, the possibility of the disease should be considered, and liver biopsy and genetic testing should be pursued.

Although BRIC rarely develops into advanced liver disease, repeated episodes can lead to a significant decline in quality of life. The main aim of treatment is to reduce the frequency of attack and prevent recurrence. Vitamins, UDCA, rifampicin, cholestyramine and corticosteroids (18–22) are currently the main pharmacologic treatments for BRIC. Various new treatment methods are constantly being proposed. Inhibition of ileal bile acid transporter can interrupt hepatoenteric circulation and reduce blood bile acid to relieve pruritus. IBAT inhibitors can be used as a non-invasive method to relieve cholestasis symptoms (23). Endoscopic nasobiliary drainage (ENBD) is a method to improve cholestasis, which can be used in BRIC patients with refractory pruritus during long-term cholestasis attacks. Molecular absorbent recirculating system (MARS) therapy is safe in the treatment of refractory pruritus in BRIC patients and can effectively reduce the biochemical indicators of cholestasis. If MARS is not successful, plasma exchange can be combined (24).

Haploinsufficiency (HI) is defined as insufficient function to maintain a wild-type phenotype in the presence of one wild-type allele and one mutant allele. The relationship between genotype and phenotype is not linear, and the specific function of a gene determines its sensitivity to dose change (25). Previous report (8) showed that different clinical symptoms in FIC patients may be related to the degree of gene mutation and heterozygous mutations are more common in BRIC1, indicates that HI may play a role in FIC, which deserves further investigation and discussion.

## Conclusion

The severity of disease caused by the *ATP8B1* mutation is related to the severity of the *ATP8B1* mutation and the function of the residual *ATP8B1*. However, the severity is not completely proportional to the reduced expression levels of *ATP8B1* protein. In the future, a reliable *ATP8B1* protein function detection method is needed to evaluate the severity and prognosis of disease. Research has found that human peripheral blood monocyte-derived macrophages (HMDMs) can be used evaluate *ATP8B1* function (15, 26).

New treatments are being proposed continually. *ATP8B1* defects lead to cystic fibrosis transmembrane conductance regulator (CFTR) downregulation (27). Targeted damage to the plasma membrane of *ATP8B1* caused by I661T, the most common *ATP8B1* disease mutation in European patients, was proven to be resolved by the CFTR corrector (28). Protein homeostasis regulators therefore represent a possible therapeutic strategy. Hepatocyte transplantation has been shown to correct pathological changes in PFIC3 model mice (29), but no human experimental studies have been conducted to date. However, 4-phenylbutyrate can be used as a chemical partner of the fold-defect variant BSEP and has been successfully used in BRIC2 (30). In addition, whether regulatory factors are available that can be used evaluate and regulate disease phenotypes still needs to be further explored.

Gene therapy corrects faulty genes that cause diseases to develop. The use of viral vectors for gene therapy has been proposed in PFIC3 (31, 32), and this methodology may be applied in the treatment of patients with clinically specific mutations of PFIC and BRIC in the future. Compensatory modified U1 snRNA is complementary to the mutated donor splicing site and exhibits great therapeutic potential as a new therapeutic strategy for *ATP8B1* defects as well as other genetic diseases (33, 34). In the future, how to regulate the expression of pathogenic genes and provide targeted individualized treatment for the functional defects of pathogenic proteins, such as gene therapy with viral vectors and protein homeostasis regulators, are worthy of our efforts.

## Data Availability Statement

The original contributions presented in the study are included in the article/Supplementary Material, further inquiries can be directed to the corresponding author.

## Author Contributions

HB drafted the script and conducted literature research. BC carried out critical revision of the manuscript for important intellectual content and final approval of the manuscript. Y-LL, DL, and CZ reviewed literature and summarized information. All authors contributed to the article and approved the submitted version.

## Conflict of Interest

The authors declare that the research was conducted in the absence of any commercial or financial relationships that could be construed as a potential conflict of interest.

## Publisher’s Note

All claims expressed in this article are solely those of the authors and do not necessarily represent those of their affiliated organizations, or those of the publisher, the editors and the reviewers. Any product that may be evaluated in this article, or claim that may be made by its manufacturer, is not guaranteed or endorsed by the publisher.

## Abbreviations

ALP: alkaline phosphatase
ALT: alanine aminotransferase
AST: aspartate aminotransferase
*ATP8B1*: ATPase class I, type 8B, member 1
BRIC: benign recurrent intrahepatic cholestasis
BSEP: bile salt export pump
CFTR: cystic fibrosis transmembrane conductance regulator
CT: computed tomography
DBIL: direct bilirubin
ENBD: endoscopic nasobiliary drainage
FIC1: familial intrahepatic cholestasis 1
FXR: farnesoid X receptor
GGT: gamma-glutamyltransferase
HE: hematoxylin-eosin
HI: haploinsufficiency
HMDMs: human peripheral blood monocyte-derived macrophages
ICP: intrahepatic cholestasis of pregnancy
MARS: molecular absorbent recirculating system
MRCP: magnetic resonance cholangiopancreatography
PBC: primary biliary cholangitis
PS: phosphatidylserine
PFIC: progressive familial intrahepatic cholestasis
TBA: total bile acid
TBIL: total bilirubin
UDCA: ursodeoxycholic acid.
