# Supplementary material for: Case Report: A Rare Heterozygous ATP8B1 Mutation in a BRIC1 Patient: Haploinsufficiency?
Source: Front Med (Lausanne). 2022 Jun 16;9:897108. doi: 10.3389/fmed.2022.897108 (PMC9243653; doi:10.3389/fmed.2022.897108)
Supplement: Supplementary file 1 [file Table_1.pdf]

Supplementary table 1. Molecular Genetic test Report.

Name	***	Age	17 years old
Sampling Date	2020-05-08	Gender	Male
Report Date	2020-06-01	Sample Type	EDTA Whole Blood
Inspection department	The First Affiliated Hospital of China Medical University	Test Item	Focus-hepatopathy

Genetic test results

Genomic DNA was extracted from the samples submitted by the subjects, and the sequencing data were analyzed by constructing genomic DNA library, enriching target DNA fragments, Illumina sequencing and bioinformatics software.

No clear pathogenic changes were observed in this sample within the detection range, but the existence of undetected variation outside the detection range cannot be ruled out.

High-throughput sequencing parameters captured in the target area

Sample number	The coverage of the target area	Average depth of target area	The 20X coverage of the target area
QX2000346401	99.90%	117.310	99.60%

Other mutations of unknown clinical significance

**Gene**	**Variation information**	**Normal person carrying frequency gnomAD/ExAC**	**Homozygous/Heterozygous**	**Sequencing depth**<br>**Variation ratio**	**Transcription version**<br>**Gene subregion**	**Genetic disease information**
AHI1	c.3173C>G<br>chr6-135644455<br>p.A1058G	0<br>0.000009	Het	48.0/56<br>0.54	NM_01765 1.4<br>exon24	1. Joubert syndrome type 3 (AR)
AMACR	c.888G>A<br>chr5-33989459<br>p.P296P	0<br>0	Het	81.0/73<br>0.47	NM_01432 4.5<br>exon5	1. Congenital bile acid synthesis disorder type 4 (AR)<br>2. A deficiency of α -formyl-coA racemate (AR)

ATP8B1	c.2663C>A<br>chr18-55328450<br>p.T888K	0<br>0	Het	96.0/80<br>0.45	NM_00560 3.4<br>exon22	1. ICP-1(AD)<br>2. PFIC-1(AR)<br>2. BRIC-1(AR)
ERCC6	c.2645A>G<br>chr10-50681587<br>p.Y882C	0.000065<br>0.000016	Het	34.0/19<br>0.36	NM_00012 4.2<br>exon14	1. Cockayne syndrome type B (AR)<br>2. Lung cancer (AD ， SMu)<br>3. Brain-eye-facial skeletal syndrome type 1 (AR)<br>4. Uv sensitivity syndrome Type 1(AR)<br>5. Age related macular degeneration type 5 (-)<br>6 、 De Sanctis-Cacchione syndrome(AR)
FASTKD2	c.-51+8G>T<br>chr2-207630386<br>splice	0<br>0	Het	3.0/7<br>0.7	NM_01492 9.3<br>intron1	1. Defective mitochondrial complex IV (AR， Mi)<br>2. Combined oxidative phosphorylation defect type 44 (AR)
GPC4	c.1469-4C>T<br>chrX-132437101<br>splice	0<br>0.000011	Hem	0.0/36<br>1	NM_00144 8.2<br>intron8	1. Keipert syndrome(XR)
LRBA	c.6047-3T>C<br>chr4-151518270<br>splice	0<br>0.000117	Het	14.0/8<br>0.36	NM_00672 6.4<br>intron38	2. Common variant immune deficiency type 8 with autoimmune disease (AR)
TPI1	c.631+3G>A<br>chr12-6979303<br>splice	0<br>0.000016	Het	32.0/25<br>0.44	NM_00036 5.5<br>intron6	1. Propanose phosphate isomerase deficiency (AR)
UNC13D	c.2588G>A<br>chr17-73827216<br>p.G863D	0.000194<br>0.000358	Het	74.0/47<br>0.39	NM_19924 2.2<br>exon27	1. Familial hemophagocytic histiocytosis type 3 (AR)

List of Genetic Tests

**Congenital hepatic fibrosis**						**49**
AHI1	*ALMS1*	*ARL13B*	*ARL6*	*B9D1*	*B9D2*	*BBIP1*
BBS1	*BBS10*	*BBS12*	*BBS2*	*BBS4*	*BBS5*	*BBS7*
BBS9	*CC2D2A*	*CEP290*	*CEP41*	*CPLANE1*	*CSPP1*	*DNAJB11*
GANAB	*IFT27*	*INPP5E*	*IQCB1*	*KIAA0586*	*LZTFL1*	*MKS1*
NPHP1	*NPHP3*	*OFD1*	*PKD1*	*PKD2*	*PKHD1*	*RPGRIP1L*
SDCCAG8	*TCTN1*	*TCTN2*	*TMEM107*	*TMEM138*	*TMEM216*	*TMEM231*
TMEM237	*TMEM67*	*TRAF3IP1*	*TRIM32*	*TTC8*	*WDPCP*	*WDR19*
**Wilson disease**						**1**
ATP7B						
**Cholestatic liver disease**						**16**
ABCB11	*ABCB4*	*ABCD3*	*ACOX2*	*AKR1D1*	*AMACR*	*ATP8B1*
CYP7B1	*HSD3B7*	*JAG1*	*NOTCH2*	*NR1H4*	*SLC10A2*	*TJP2*
VIPAS39	*VPS33B*					
**Mitochondrial liver disease**						**70**
ATP5F1A	*ATP5F1E*	*ATPAF2*	*BCS1L*	*BOLA3*	*COA8*	*COX10*
COX14	*COX20*	*COX6B1*	*COX8A*	*CYC1*	*DGUOK*	*ECHS1*
FASTKD2	*FBXL4*	*FOXRED1*	*ISCA2*	*LYRM7*	*MGME1*	*MPC1*
MPV17	*MRM2*	*NDUFA1*	*NDUFA10*	*NDUFA11*	*NDUFA12*	*NDUFA2*
NDUFA4	*NDUFA9*	*NDUFAF1*	*NDUFAF2*	*NDUFAF3*	*NDUFAF4*	*NDUFAF5*
NDUFAF6	*NDUFB11*	*NDUFB3*	*NDUFB9*	*NDUFS1*	*NDUFS2*	*NDUFS3*
NDUFS4	*NDUFS6*	*NDUFS7*	*NDUFS8*	*NDUFV1*	*NDUFV2*	*NFS1*
NUBPL	*PCK2*	*PET100*	*POLG*	*POLG2*	*RRM2B*	*SCO1*
SLC25A4	*SUCLA2*	*SUCLG1*	*TACO1*	*TFAM*	*TK2*	*TMEM126B*
TTC19	*TWNK*	*UQCC2*	*UQCC3*	*UQCRB*	*UQCRC2*	*UQCRQ*

**Glucose metabolic disease**						**38**
AGL	*ALDOA*	*ARSB*	*CTSA*	*ENO3*	*FBP1*	*G6PC*
GAA	*GALE*	*GALK1*	*GALNS*	*GALT*	*GBE1*	*GLB1*
GNS	*GUSB*	*GYG1*	*GYS1*	*GYS2*	*HGSNAT*	*HYAL1*
IDS	*IDUA*	*LDHA*	*NAGLU*	*PFKM*	*PGAM2*	*PGM1*
PHKA1	*PHKA2*	*PHKB*	*PHKG2*	*PRKAG2*	*PYGL*	*PYGM*
SGSH	*SLC37A4*	*TPI1*				
**Defective glycoprotein synthesis**						**40**
ALG1	*ALG11*	*ALG12*	*ALG13*	*ALG2*	*ALG3*	*ALG6*
ALG8	*ALG9*	*B4GALT1*	*CCDC115*	*COG1*	*COG2*	*COG4*
COG5	*COG6*	*COG7*	*COG8*	*DDOST*	*DOLK*	*DPAGT1*
DPM1	*DPM2*	*DPM3*	*MGAT2*	*MOGS*	*MPDU1*	*MPI*
NGLY1	*PMM2*	*RFT1*	*SLC35A1*	*SLC35A2*	*SLC35C1*	*SRD5A3*
SSR4	*STT3A*	*STT3B*	*TMEM165*	*TMEM199*		
**Aminoacidopathy**						**53**
ABAT	*ABCG5*	*ADA*	*ADK*	*AGA*	*AHCY*	*AMT*
ASL	*ASS1*	*CBS*	*COQ2*	*CPS1*	*CPT1A*	*CPT2*
CTNS	*CYP7A1*	*DNAJC19*	*ETFA*	*ETFB*	*ETFDH*	*FAH*
GCDH	*GCH1*	*GCSH*	*GLDC*	*GNMT*	*GSTZ1*	*HADH*
HMGCL	*HMGCS2*	*HPD*	*MAT1A*	*MCCC1*	*MCCC2*	*MMAA*
MMAB	*MMACHC*	*MMUT*	*NAGA*	*OTC*	*PC*	*PCBD1*
PCCA	*PCCB*	*PDSS2*	*PEPD*	*PKLR*	*SLC22A5*	*SLC25A13*
SLC25A15	*SLC25A20*	*SLC7A7*	*TAT*			
**Hemochromatosis**						**7**
BMP2	*FTH1*	*HAMP*	*HFE*	*HJV*	*SLC40A1*	*TFR2*
**Porphyrinopathy**						**8**
ALAD	*ALAS2*	*CPOX*	*FECH*	*HFE*	*HMBS*	*UROD*
UROS						
**Abnormal metabolism of cholochrome**						**4**
ABCC2	*SLCO1B1*	*SLCO1B3*	*UGT1A1*			

**Peroxisomal disorders**						**17**
ABCD1	*ACOX1*	*HSD17B4*	*PEX1*	*PEX10*	*PEX11B*	*PEX12*
PEX13	*PEX14*	*PEX16*	*PEX19*	*PEX2*	*PEX26*	*PEX3*
PEX5	*PEX6*	*PEX7*				
**Polycystic liver disease**						**4**
ALG8	*LRP5*	*PRKCSH*	*SEC63*			
**Complex oxidative phosphorylation deficiency**						**23**
AARS2	*AIFM1*	*ATP5F1A*	*C12orf65*	*C1QBP*	*ELAC2*	*FARS2*
GFM1	*LYRM4*	*MRPL3*	*MRPL44*	*MRPS16*	*MRPS22*	*MRPS7*
MTFMT	*PNPT1*	*RMND1*	*SFXN4*	*TARS2*	*TRMT10C*	*TSFM*
TUFM	*VARS2*					
**Glucolipid metabolic disease**						**4**
GBA	*NPC1*	*NPC2*	*SMPD1*			
