# Supplementary figures and images for: Case Report: A Rare Heterozygous ATP8B1 Mutation in a BRIC1 Patient: Haploinsufficiency?
Source: Front Med (Lausanne). 2022 Jun 16;9:897108. doi: 10.3389/fmed.2022.897108 (PMC9243653; doi:10.3389/fmed.2022.897108)

**Supplementary Figure 1.** Pedigree of the patient with variations in ATP8B1.


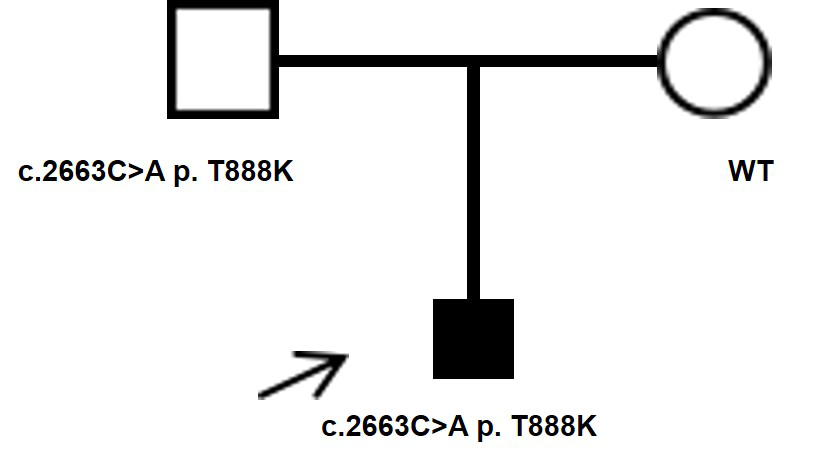

Supplement: Supplementary file 2 [file Data_Sheet_1.docx]
